# Predictive value of the fibrinogen-to-albumin ratio for hemorrhagic transformation following intravenous thrombolysis in ischemic stroke: a retrospective propensity score-matched analysis

**DOI:** 10.3389/fneur.2025.1465508

**Published:** 2025-05-01

**Authors:** Xiao-Yu Liu, Hui-Yang Jia, Gang Wang

**Affiliations:** ^1^Department of Radiology, The First Affiliated Hospital of Jinzhou Medical University, Jinzhou, China; ^2^Department of Neurology, Panjin Central Hospital, Jinzhou Medical University, Panjin, China

**Keywords:** ischemic stroke, intravenous thrombolysis, hemorrhagic transformation, fibrinogen, albumin

## Abstract

**Objective:**

This study aimed to explore the factors associated with hemorrhagic transformation (HT) in acute ischemic stroke patients after intravenous thrombolysis (IVT), with a specific focus on the relationship with the post-thrombolysis fibrinogen-to-albumin ratio (FAR).

**Methods:**

The clinical records of 569 acute ischemic stroke (AIS) patients admitted to our department from 2020 to 2023 were retrospectively analyzed. All eligible patients were stratified into HT and non-HT (NHT) groups. Propensity score matching (PSM) was performed between the two groups. Receiver operating characteristic (ROC) curves were used to assess the predictive performance of the FAR, determining the optimal predictive value.

**Results:**

Ultimately, 142 patients were included, with 71 in the HT group and 71 in the NHT group. After propensity score matching, a significant association was observed between the FAR and HT (*OR* = 1.40, 95% CI, 1.187–1.645; *p* <0.001). The ROC curve analysis indicated that the FAR predicted HT after intravenous thrombolysis, with an area under the curve (AUC) value of 0.751 (95% CI, 0.669–0.831; *p* <0.001) and an optimal cutoff value of 0.0918. The corresponding sensitivity and specificity were 78.9 and 60.9%, respectively.

**Conclusion:**

In ischemic stroke patients undergoing IVT, the FAR may serve as a promising biochemical marker for predicting HT following treatment.

## 1 Introduction

In 2022, the World Stroke Organization (WSO) highlighted in its report (1) that stroke remains the second leading cause of death worldwide, with acute ischemic stroke (AIS) accounting for 87% of all stroke cases. Currently, evidence-based management of AIS involves intravenous thrombolysis and/or mechanical thrombectomy within therapeutic time windows. Among these approaches, intravenous thrombolytic therapy with recombinant tissue plasminogen activator (rt-PA) is the preferred method for treating ischemic strokes, significantly improving neurological prognosis (2). However, hemorrhagic transformation (HT) following AIS recanalization is one of the most clinically consequential complications (3), often leading to unfavorable outcomes. The incidence of HT varies from high to low (4–6). Previous research suggests that HT may be somewhat promoted by early blood–brain barrier disruption (7, 8), and this further exacerbates neurological damage in patients and leads to poor prognosis. These findings highlight the critical need for early risk stratification tools and novel neuroprotective strategies.

One factor contributing to the blood–brain barrier dysregulation is fibrinogen (FIB). Elevated levels of fibrinogen may also increase the risk of stroke, leading to poorer outcomes (9). The antioxidant, anti-apoptotic, and anti-inflammatory properties of albumin (ALB) have neuroprotective functions, helping maintain normal blood–brain barrier permeability. Compelling data from multicenter cohorts have shown (10) that hypoalbuminemia is associated with poor prognosis in AIS patients. Therefore, the fibrinogen-to-albumin ratio (FAR), which integrates both anti-inflammatory and pro-inflammatory indicators, has been developed and may be related to the occurrence of HT to a certain extent. It could potentially serve as a predictor of bleeding after thrombolysis. This novel systemic inflammation index has already established strong prognostic validity in oncology (11), cardiovascular medicine (12), and respiratory pathophysiology (13). Nevertheless, significant knowledge gaps remain regarding the predictive value of the FAR for post-thrombolysis HT in neurovascular contexts, particularly in terms of optimal threshold determination and evidence-based cutoff values. We hypothesize that the FAR could serve as a prognostic predictor of HT. Therefore, the goal of this research was to investigate the relationship between the FAR and HT.

## 2 Materials and methods

### 2.1 Study population

A retrospective cohort analysis was conducted on ischemic stroke patients who received alteplase (rt-PA) thrombolysis at our hospital between December 2020 and December 2023. All patients met the diagnostic criteria and indications for AIS outlined in the guidelines (14). The exclusion criteria included the following: (1) Intravascular therapy after intravenous thrombolysis (including intravascular thrombolysis and arterial thrombolysis); (2) missing or incomplete clinical and laboratory data; and (3) active malignancies, immune-mediated disorders, or acute infectious diseases. The study involving human participants was reviewed and approved by the Ethics Committee of the First Affiliated Hospital of Jinzhou Medical University. Due to the retrospective nature of this study, the First Affiliated Hospital of Jinzhou Medical University was exempt from the requirement for written consent.

### 2.2 Data collection

Data were retrospectively collected for each patient before intravenous thrombolysis and were immediately recorded following the clinical and radiological diagnosis of AIS. Baseline clinical characteristics included sex (male/female), age (years), smoking status, and alcohol use history. Clinical data included the National Institutes of Health Stroke Scale (NIHSS) score, systolic and diastolic blood pressure at admission, and medical history, including hypertension, coronary artery disease, diabetes, and bleeding diathesis. Laboratory data obtained from blood tests conducted before intravenous thrombolysis included the following components: white blood cells (WBC), monocytes (MN), eosinophils (EO), basophils (BA), neutrophils (NE), lymphocyte**s** (LY), total protein (TP), albumin (ALB), globulin (GLB), fibrinogen (FIB), triglyceride**s** (TG), cholesterol (CHO), low-density lipoprotein (LDL), and high-density lipoprotein (HDL). Stroke severity was assessed by a trained neurologist upon admission using the NIHSS score (15). The FAR was calculated using the formula: FAR = FIB/ALB.

### 2.3 Dosage and treatment regimen

A dose of 0.9 mg/kg (with a maximum of 90 mg) was administered over 60 min, with 10% of the total dose given as a bolus within 1 min.

### 2.4 Primary clinical outcome

The main clinical outcome was HT, which was confirmed through CT or MRI. HT subtypes were classified according to the European Cooperative Acute Stroke Study II (ECASS II) criteria (16) into two categories: parenchymal hemorrhage (PH) and hemorrhagic infarction (HI).

### 2.5 Statistical method

Statistical analyses were performed using SPSS 26.0 software (IBM, Armonk, NY). Continuous data with normal distribution were expressed as mean ± standard deviation (SD), while continuous data with non-normal distribution were expressed as median and quartile spacing [M (P25, P75)]. Intergroup comparisons were performed using Student’s ***t***-test for parametric data and the Mann–Whitney U-test for non-parametric distributions. Count data were expressed as frequency and percentage [*n* (%)], and comparisons of categorical variables between the groups were performed using either a chi-squared test or Fisher’s exact test, as appropriate. Data normality was verified using the Kolmogorov**–**Smirnov test. A two-tailed α-level of 0.05 was considered to indicate statistical significance.

The patients were prospectively stratified into HT and non-HT (NHT) groups. Following the identification of HT-associated predictors, 1:1 propensity score matching (PSM) was implemented with a caliper width of 0.02 to mitigate confounding biases, thereby achieving quasi-randomized trial equivalency in baseline characteristics. Receiver operating characteristic (*ROC*) curves were used to determine the area under the curve (AUC), sensitivity, and specificity of the FAR in predicting HT after intravenous thrombolysis. The optimal cutoff value for the FAR was determined by calculating the maximum Youden’s index.

## 3 Results

**Figure 1:**
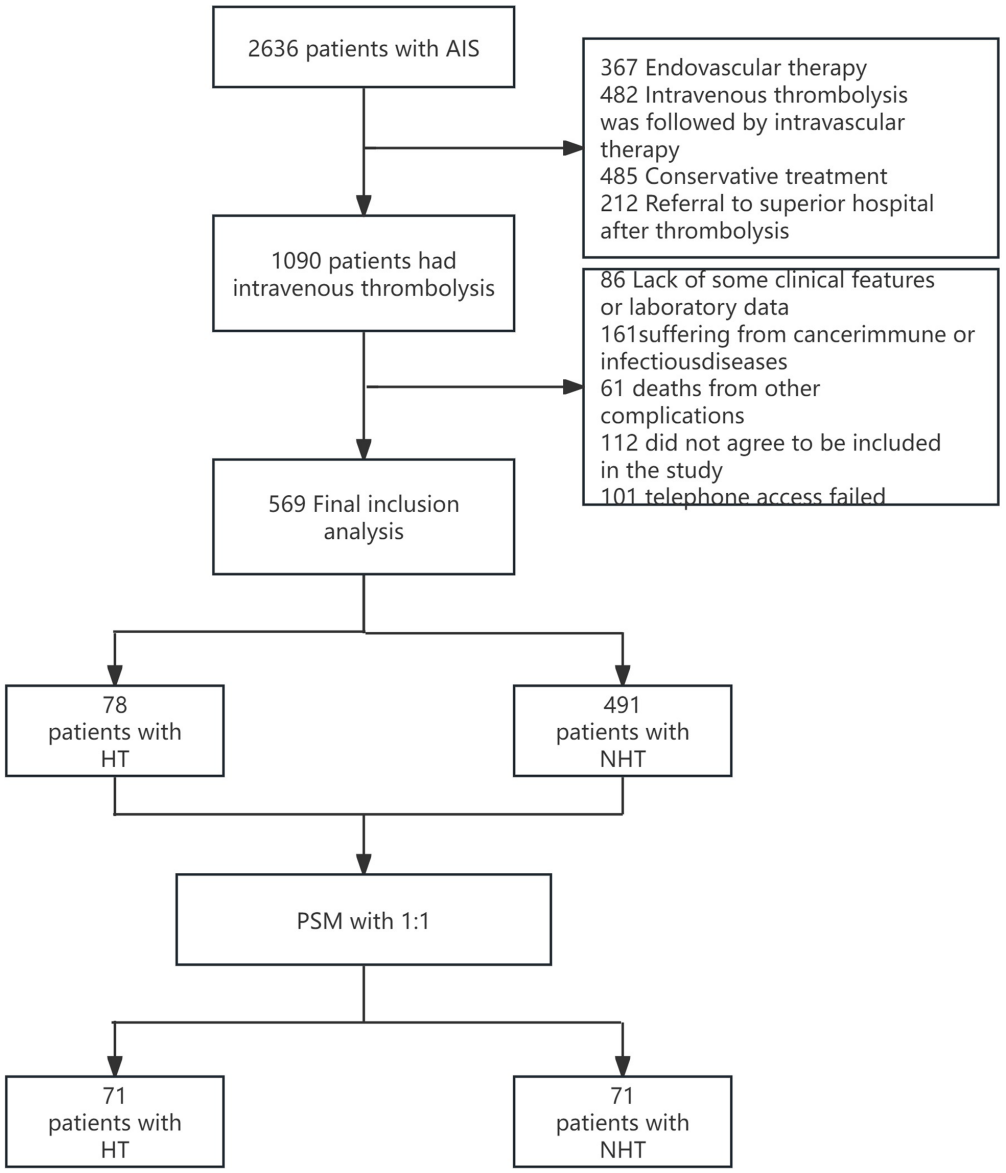
Flow diagram. AIS, acute ischemic stroke; HT, Hemorhagicnon-Hemorrhagic transformation; PSM, Propensity score matching.

A total of 2,636 AIS patients were initially screened. After applying the predefined exclusion criteria and accounting for missing data, 1,546 patients were excluded, leaving 569 eligible participants for preliminary analysis. Subsequently, PSM analysis was performed, and 142 patients were selected for statistical analysis. There were 71 cases in the HT group and 71 cases in the NHT group. A detailed flowchart of this process is displayed in Figure 1.

In this study, 78 patients (13.7%) developed HT after thrombolysis. The baseline characteristics and laboratory parameters are presented in Table 1. Statistically significant differences between the two groups were observed in the following variables: age, coronary artery disease, NIHSS score, RBC count, FIB level, ALB level, and FAR. Consequently, when using the FAR as a predictive biomarker for early post-thrombolysis HT, potential confounding factors must be taken into account. This means that, both in research and clinical practice, it is essential to not only focus on the FAR but also to adjust for variables such as age, history of coronary artery disease, and early NIHSS score in order to enhance the predictive accuracy of HT risk assessment.

**Table 1:** Baseline characteristics and laboratory results of patients with HT.

Variable	HT (*N* = 78)	NHT (*N* = 491)	*p-*value
Male, *n* (%)	55 (70.5%)	336 (68.4%)	0.713
Age, median (P25, P75)	74 (69, 80)	67 (59, 75)	<0.001*
Hypertension, *n* (%)	46 (58.9%)	291 (59.2%)	0.961
Coronary heart disease, *n* (%)	20 (25.6%)	72 (14.6%)	0.014*
Diabetes, *n* (%)	16 (20.5%)	123 (25.0%)	0.386
Smoking, *n* (%)	40 (51.2%)	221 (45.0%)	0.302
Alcoholism, *n* (%)	27 (34.6%)	167 (34.0%)	0.917
Bleeding gums, *n* (%)	8 (10.2%)	47 (9.5%)	0.849
SBP, mmHg, median (P25, P75)	173 (147, 194)	167 (145, 186)	0.086
DBP, mmHg, median (P25, P75)	92 (85, 105)	94 (84, 105)	0.715
Blood glucose, mmol/L, median (P25, P75)	98.1 (6.1, 10.6)	8.2 (6.7, 10.5)	0.394
NIHSS score, median (P25, P75)	14.5 (9.75, 18)	10.0 (5, 14)	<0.001*
TOAST classification			0.629
Intracranial atherosclerosis	50 (64.1%)	276 (56.2%)	
Cardioembolism	13 (16.6%)	97 (19.7%)	
Arterial occlusive type	10 (12.8%)	80 (16.3%)	
Unexplained type	5 (6.4%)	38 (7.7%)	
Platelet, mean ± SD	4.46 ± 0.52	4.61 ± 0.51	0.021*
WBC, median (P25, P75)	8.27 (6.57, 9.70)	7.71 (6.39, 9.98)	0.575
Monocyte, median (P25, P75)	0.42 (0.33, 0.58)	0.42 (0.32, 0.54)	0.837
Eosinophil, median (P25, P75)	0.16 (0.08, 0.37)	0.14 (0.07, 0.50)	0.802
Basophil, median (P25, P75)	0.05 (0.03, 0.26)	0.04 (0.03, 0.20)	0.166
Neutrophil, median (P25, P75)	5.55 (4.27, 6.27)	5.09 (3.81, 6.68)	0.160
Lymphocyte, median (P25, P75)	1.81 (1.26, 2.47)	1.88 (1.33, 2.57)	0.208
Fibrinogen, median (P25, P75)	3.80 (2.92, 4.73)	3.45 (3.00, 3.92)	0.011*
Total protein, median (P25, P75)	71.3 (67.5, 74.1)	71.8 (67.8, 75.7)	0.498
Albumin, median (P25, P75)	40.8 (38.7, 42.8)	41.9 (39.6, 43.7)	0.050 *
FAR, median (P25, P75)	0.096 (0.0807, 0.116)	0.081 (0.072, 0.095)	<0.001*
Globulin, median (P25, P75)	30.9 (28.2, 32.8)	30.9 (27.2, 33.2)	0.617
Triglyceride, median (P25, P75)	1.35 (0.80, 1.77)	1.43 (1.00, 2.12)	0.071
Cholesterol, median (P25, P75)	4.77 (4.12, 5.34)	4.71 (3.99, 5.29)	0.755
Low-density lipoprotein, median (P25, P75)	2.84 (2.27, 3.28)	2.71 (2.16, 3.28)	0.463
High-density lipoprotein, median (P25, P75)	1.21 (0.96, 1.37)	1.17 (0.99, 1.35)	0.889

SBP, systolic blood pressure; DBP, diastolic blood pressure; NIHSS, National Institutes of Health Stroke Scale, WBC, white blood cells; NIHSS, National Institutes of Health Stroke Scale; FAR, fibrinogen-to-albumin ratio. Differences marked with * are statistically significant (*p* < 0.05).

A total of 142 patients were selected for statistical analysis using propensity score matching, with 71 patients assigned to the HT group and 71 assigned to the NHT group. No significant differences were observed between the two groups regarding confounding factors for HT, particularly age, coronary heart disease, initial NIHSS score, RBC count, FIB level, and ALB level. However, the FAR demonstrated a statistically significant difference between the two groups (OR, 1.40; 95% CI, 1.187–1.645; *p* < 0.001) (Table 2).

**Table 2:** Baseline characteristics and laboratory results of patients with HT after propensity score matching.

Variable	HT (*N* = 71)	NHT (*N* = 71)	OR(95% CI)	*P*-value
Male, *n* (%)	49 (69.0%)	21 (29.5%)	NA	0.855
Age, median (P25, P75)	72 ± 7	72 ± 8	NA	0.504
Hypertension, *n* (%)	40 (56.3%)	37 (52.1%)	NA	0.613
Coronary heart disease, *n* (%)	16 (22.5%)	19 (26.7%)	NA	0.559
Diabetes, *n* (%)	16 (22.5%)	19 (26.7%)	NA	0.559
Smoking, *n* (%)	34 (47.8%)	32 (45.2%)	NA	0.736
Alcoholism, *n* (%)	23 (32.3%)	22 (30.9%)	NA	0.857
Bleeding gums, *n* (%)	8 (11.2%)	7 (9.8%)	NA	0.785
SBP, mmHg, median (P25, P75)	172 ± 34	166 ± 35	NA	0.288
DBP, mmHg, median (P25, P75)	91 (84, 104)	93 (85, 103)	NA	0.956
Blood glucose, mmol/L, median (P25, P75)	8.2 (6.6, 11.2)	8.2 (6.4, 10.6)	NA	0.579
NIHSS score, median (P25, P75)	14 (7, 18)	12 (7, 19)	NA	0.686
TOAST classification				0.898
Intracranial atherosclerosis	45 (63.4%)	49 (69.0%)		
Cardioembolism	11 (15.5%)	9 (12.7%)		
Arterial occlusive type	10 (14.1%)	8 (11.3%)		
Unexplained type	5 (7.0%)	5 (7.0%)		
RBC, median (P25, P75)	4.46 ± 0.52	4.41 ± 0.60	NA	0.541
WBC, median (P25, P75)	8.26 (6.61, 9.76)	7.18 (5.91, 8.86)	NA	0.092
Monocyte, median (P25, P75)	0.41 (0.33, 0.58)	0.41 (0.30, 0.50)	NA	0.299
Eosinophil, median (P25, P75)	0.16 (0.06, 0.40)	0.13 (0.07, 1.00)	NA	0.906
Basophil, median (P25, P75)	0.05 (0.03, 0.30)	0.05 (0.02, 0.30)	NA	0.802
Neutrophil, median (P25, P75)	5.55 (4.24, 6.85)	4.79 (3.60, 6.55)	NA	0.256
Lymphocyte, median (P25, P75)	1.81 (1.30, 2.52)	1.76 (1.17, 2.27)	NA	0.237
Fibrinogen, median (P25, P75)	3.70 (2.83, 4.74)	3.48 (2.78, 4.03)	NA	0.124
Total protein, median (P25, P75)	71.3 (67.6, 73.8)	70.7 (65.6, 74.2)	NA	0.412
Albumin, median (P25, P75)	40.7 (38.7, 42.1)	41.7 (38.8, 43.5)	NA	0.427
FAR, median (P25, P75)	0.101 (0.093, 0.117)	0.083 (0.069, 0.100)	1.401 (1.187–1.654)	<0.001*
Globulin, median (P25, P75)	30.4 ± 3.66	29.52 ± 4.57	NA	0.109
Triglyceride, median (P25, P75)	1.43 (0.90, 1.77)	1.29 (0.99, 1.76)	NA	0.576
Cholesterol, median (P25, P75)	4.67 ± 0.97	4.51 ± 1.3	NA	0.407
Low-density lipoprotein, median (P25, P75)	2.79 (2.27, 3.24)	2.79 (2.16, 3.30)	NA	0.846
High-density lipoprotein, median (P25, P75)	1.17 ± 0.24	1.18 ± 0.27	NA	0.829

SBP, systolic blood pressure; DBP, diastolic blood pressure; NIHSS, National Institutes of Health Stroke Scale, WBC, white blood cells; NIHSS, National Institutes of Health Stroke Scale; FAR, fibrinogen-to-albumin ratio. Differences marked with * are statistically significant (*p* < 0.05).

**Figure 2:**
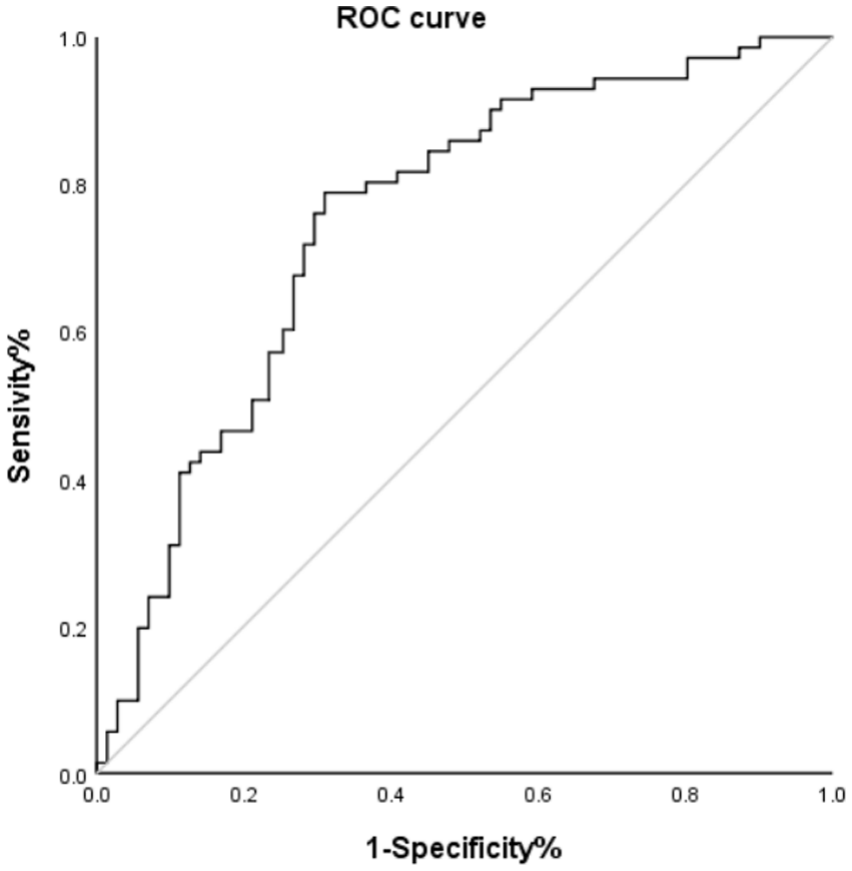
ROC curve of HT and FAR. AUC, area under the curve.

The ROC curve shown in Figure 2 indicates that the FAR has significant predictive value for patient prognosis. The area under the curve (AUC) was 0.75 (95% CI, 0.669–0.831; *p*<0.001), with an optimal cutoff value of 0.0918, yielding a sensitivity of 78.9% and a specificity of 69.0% for the FAR.

**Figure 3:**
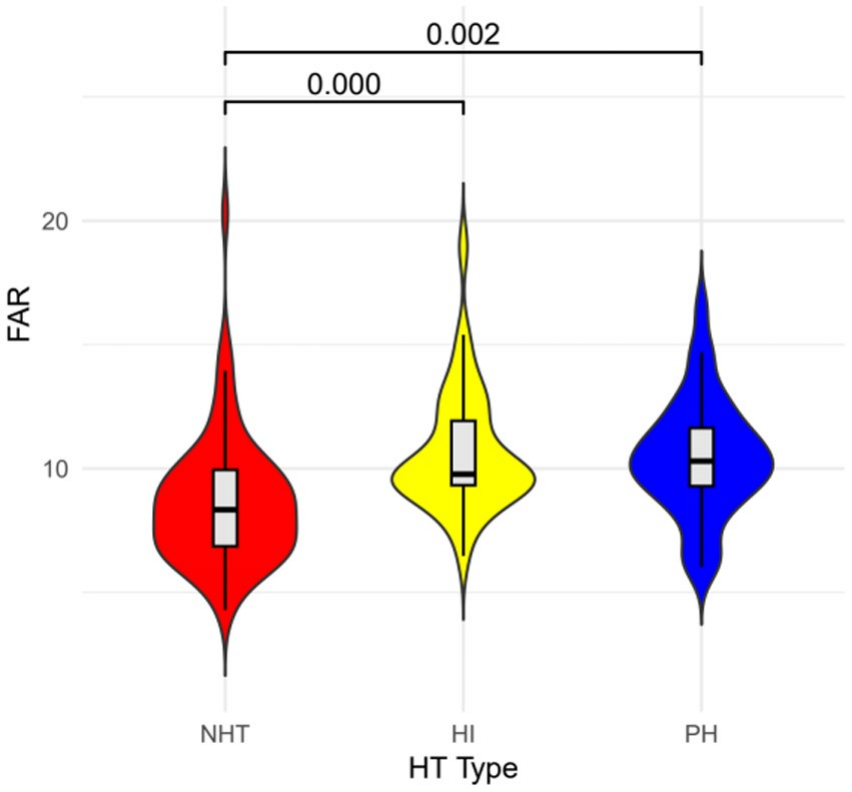
FAR violin plot. FAR, fibrinogen-to-albumin ratio; Hl, hemorrhagic infarct; HT, hemorrhagic transformation; PH, parenchymal hematoma.

Of the 71 HT patients, 36 were diagnosed with HI and 35 with PH. Figure 3 illustrates the distribution of NHT and the FAR within HT classification. The FAR in the HI group was significantly higher than that in the NHT group (*p* <0.001), and similarly, the FAR in the PH group was also elevated compared to the NHT group (*p* = 0.002).

Using the optimal cutoff value of 0.0918 for the FAR, the patients were stratified into a high-FAR cohort (≥ 0.0918) and a low-FAR cohort (< 0.0918). Table 3 summarizes the clinical and laboratory characteristics between these two cohorts. After adjusting for the aforementioned confounding factors, we observed that a high FAR was independently associated with HT (OR, 3.453; 95% CI, 1.539–7.743; *p* = 0.003).

**Table 3:** Baseline characteristics and laboratory results of patients in the low (≤0.0918) and high (>0.0918) FAR groups.

Variable	Univariate analysis		Multivariate analysis	
	OR(95% CI)	*P-*value	OR(95% CI)	*P*-value
Male, *n* (%)	1.175 (0.800–1.725)	0.411	NA	
Age, median (P25, P75)	1.034 (1.017–1.052)	<0.001*	0.993 (0.964–1.020)	0.617
Hypertension, *n* (%)	0.973 (0.481–1.881)	0.920	NA	
Coronary heart disease, *n* (%)	1.290 (0.810–2.055)	0.283	NA	
Diabetes, *n* (%)	1.203 (0.803–1.800)	0.370	NA	
Smoking, *n* (%)	1.035 (0.727–1.473)	0.849	NA	
Alcoholism, *n* (%)	0.822 (0.565–1.1978)	0.307	NA	
Bleeding gums, *n* (%)	0.772 (0.415–1.438)	0.415	NA	
SBP, mmHg, median (P25, P75)	0.998 (0.993–1.004)	0.515	NA	
DBP, mmHg, median (P25, P75)	1.002 (0.992–1.012)	0.723	NA	
Blood glucose, mmol/L, median (P25, P75)	1.030 (0.986–1.077)	0.184	NA	
NIHSS score, median (P25, P75)	1.037 (1.014–1.061)	0.002*	1.003 (0.966–1.042)	0.856
TOAST classification		0.141	NA	
Intracranial atherosclerosis	1.278 (0.386–3.061)			
Cardioembolism	1.331 (0.502–3.517)			
Arterial occlusive type	1.363 (0.494–3.756)			
Unexplained type	1.094 (0.303–4.016)			
RBC, median (P25, P75)	0.491 (0.350–0.690)	<0.001*	0.978 (0.554–1.724)	0.938
WBC, median (P25, P75)	1.040 (0.982–1.101)	0.183	NA	
Monocyte, median (P25, P75)	1.071 (0.810–1.415)	0.632	NA	
Eosinophil, median (P25, P75)	0.976 (0.849–1.121)	0.728	NA	
Basophil, median (P25, P75)	0.697 (0.289–1.683)	0.422	NA	
Neutrophil, median (P25, P75)	1.045 (0.982–1.112)	0.168	NA	
Lymphocyte, median (P25, P75)	0.930 (0.796–1.085)	0.355	NA	
Fibrinogen, median (P25, P75)	11.778 (7.608–18.232)	<0.001*	15.670 (9.220–26.632)	<0.001*
Total protein, median (P25, P75)	0.971 (0.945–0.997)	0.027*	1.006 (0.879–1.152)	0.928
Albumin, median (P25, P75)	0.753 (0.703–0.806)	<0.001*	0.692 (0.579–0.826)	<0.001*
HT, *n* (%)	3.488 (2.136–5.696)	<0.001*	3.453 (1.539–7.743)	0.003*
Globulin, median (P25, P75)	1.059 (1.024–1.096)	0.001*	1.009 (0.872–1.168)	0.904
Triglyceride, median (P25, P75)	0.901 (0.800–1.015)	0.086	NA	
Cholesterol, median (P25, P75)	1.089 (0.939–1.262)	0.258	NA	
Low-density lipoprotein, median (P25, P75)	1.213 (1.006–1.470)	0.053	NA	
High-density lipoprotein, median (P25, P75)	0.491 (0.264–0.913)	0.025*	1.665 (0.676–4.099)	0.267

SBP, systolic blood pressure; DBP, diastolic blood pressure; NIHSS, National Institutes of Health Stroke Scale; WBC, white blood cells; NIHSS, National Institutes of Health Stroke Scale; FAR, fibrinogen-to-albumin ratio. Differences marked with * are statistically significant (*p* < 0.05).

## 4 Discussion

Multiple pathophysiological mechanisms contribute to the occurrence of HT in patients with AIS who receive IVT. However, previous studies have rarely focused on the prognostic potential of serum biomarkers. Our study revealed that the baseline FAR was significantly higher in patients with HT after intravenous thrombolysis compared to those without HT (*p* < 0.001). After adjusting for potential confounding variables using PSM, the FAR remained an independent predictor of HT. Our study identified the optimal threshold value of the FAR for predicting the occurrence of HT as 0.0918 (with a sensitivity of 78.9% and specificity of 69.0%). Currently, studies exploring the association between the FAR and HT after intravenous thrombolysis in stroke patients appear to be limited to those by Ruan et al. (17) and Yang et al. (18). However, both studies have methodological limitations. Although they attempted to control for confounding factors to some extent, they might still have overlooked important variables, which limits the veracity and reliability of their findings. In contrast, our study used PSM analysis to balance the effects of confounding factors and to simulate the effects of randomized controlled trials, thereby enhancing the accuracy of the results. Furthermore, our ROC curve also showed better sensitivity, specificity, and AUC values. Collectively, these findings highlight the FAR as a clinically actionable biomarker for stratifying the risk of HT in IVT-eligible AIS populations.

FIB, a dual-function protein involved in acute inflammatory responses and coagulation cascades, plays a critical role in platelet aggregation, fibrin formation, and dissolution. Elevated levels of FIB are known to disrupt the blood–brain barrier (19, 20), leading to neuroinflammation and neuronal damage. Conversely, the use of FIB-reducing treatments in AIS patients has shown promise in reducing neurological impairment and enhancing quality of life (21, 22). FIB plays a role in systemic inflammatory responses by regulating fibrin production and activating signaling pathways upon binding to microglia/macrophage receptors, thereby promoting inflammation. This, in turn, is associated with blood–brain barrier breakdown and brain injury (15, 23, 24). Mechanistically, ischemic stroke induces the upregulation of pro-inflammatory cytokines, particularly interleukin-6 (IL-6), which stimulates fibrinogen overexpression (25, 26). Concomitantly, increased levels of FIB and the activation of extracellular matrix proteolytic enzymes, particularly matrix metalloproteinases, contribute to extracellular matrix degradation and disrupt tight endothelial cell connections (26, 27). Ultimately, as a key component of the perivascular extracellular matrix, FIB modulates cerebrovascular endothelial cell permeability, thereby increasing blood–brain barrier permeability (28).

In contrast to prothrombotic biomarkers, ALB exhibits a neuroprotective effect that mitigates the risk of HT. First, ALB acts as a specific inhibitor of endothelial cell apoptosis and a major antioxidant, reducing the permeability of the blood–brain barrier. Second, it curtails the inflammatory response by suppressing the adhesion of white blood cells in microcirculation. Third, due to its high molecular weight, serum albumin decreases the blood–brain barrier permeability (29). In summary, ALB plays a vital role in the protection of the nervous system (30, 31). Yang et al. (18) proposed that ALB serves as a protective factor against HT following intravenous thrombolysis (OR, 0.907; 95% confidence interval, 0.859–0.957; *p* < 0.001). Multicenter studies have shown that elevated ALB levels are associated with favorable nerve recovery, reduced cerebral infarction volume, and alleviated cerebral edema (32, 33).

The FAR, an emerging thromboinflammatory biomarker, has been proposed and documented (34). Mechanistically, the synergistic combination of FIB and ALB outperforms individual biomarkers in predicting post-thrombolysis HT, due to their counterregulatory biological interactions. An increase in the FAR signifies either heightened FIB levels or reduced ALB levels. The interplay between ALB and FIB may inhibit FIB activity, mitigating fibrin accumulation (35). This suppression cascade indirectly diminishes the likelihood of FIB transforming into fibrin, thereby reducing the probability of inflammatory factor release and, consequently, decreasing the risk of blood–brain barrier damage. Our results corroborate seminal findings (17), indicating that an elevated FAR before thrombolysis is independently correlated with the occurrence of HT and increases the risk of HT. A higher FAR signifies an intensified inflammatory state, leading to the breakdown of blood–brain barrier and a higher incidence of HT. Individuals with a higher FAR exhibit higher 90-day mortality rates and poorer functional recovery.

This study has several limitations that warrant consideration. First, as a retrospective single-center analysis, the design was inherently vulnerable to confirmation bias and potential selection bias, which might have affected the generalizability of the findings. Second, the FAR exhibits temporal variability, and since it was measured only once in the included studies, this might have introduced observational inaccuracies. Third, the analysis incorporated only a limited panel of laboratory parameters, potentially overlooking other clinically relevant biomarkers that could modulate outcomes. Fourth, individual variations in the FAR may arise from environmental factors and heterogeneous physiological responses, complicating the standardized interpretation of the results. Fifth, neurofunctional prognosis must also be considered. To address these limitations, it is necessary for further prospective studies to thoroughly evaluate the dynamic changes in the FAR and its predictive value for HT.

## 5 Conclusion

In conclusion, the FAR emerges as a valuable predictor of HT following intravenous thrombolysis in patients with ischemic stroke. An elevated FAR is strongly associated with an augmented risk of HT following intravenous thrombolysis.

## Data Availability

The studies involving humans were approved by the Ethics Committee of the First Affiliated Hospital of Jinzhou Medical University. The studies were conducted in accordance with the local legislation and institutional requirements. Written informed consent for participation was not required from the participants or the participants’ legal guardians/next of kin in accordance with the national legislation and institutional requirements.
